# Brain Tumor Microenvironment and Angiogenesis in Melanoma Brain Metastases

**DOI:** 10.3389/fonc.2020.604213

**Published:** 2021-01-21

**Authors:** Dimitri G. Trembath, Eric S. Davis, Shanti Rao, Evan Bradler, Angelica F. Saada, Bentley R. Midkiff, Anna C. Snavely, Matthew G. Ewend, Frances A. Collichio, Carrie B. Lee, Georgia-Sofia Karachaliou, Fatih Ayvali, David W. Ollila, Michal T. Krauze, John M. Kirkwood, Benjamin G. Vincent, Nana Nikolaishvilli-Feinberg, Stergios J. Moschos

**Affiliations:** ^1^ Departments of Pathology and Laboratory Medicine, The University of North Carolina at Chapel Hill, Chapel Hill, NC, United States; ^2^ Lineberger Comprehensive Cancer Center, The University of North Carolina at Chapel Hill, Chapel Hill, NC, United States; ^3^ University of North Carolina School of Medicine, Chapel Hill, NC, United States; ^4^ State University of New York Downstate Medical Center College of Medicine, Brooklyn, NY, United States; ^5^ Translational Pathology Laboratory, The University of North Carolina at Chapel Hill, Chapel Hill, NC, United States; ^6^ Department of Neurosurgery, The University of North Carolina at Chapel Hill, Chapel Hill, NC, United States; ^7^ Department of Medicine, The University of North Carolina at Chapel Hill, Chapel Hill, NC, United States; ^8^ Department of Surgery, The University of North Carolina at Chapel Hill, Chapel Hill, NC, United States; ^9^ Melanoma and Skin Cancer Program, University of Pittsburgh Medical Center, Pittsburgh, PA, United States

**Keywords:** melanoma brain metastases, vascular endothelial growth factor, basic fibroblast growth factor, tumor infiltrating lymphocytes, CD31, pericyte, peritumoral edema, intratumoral hemorrhage

## Abstract

**Background:**

High tumor-infiltrating lymphocytes (TILs) and hemorrhage are important prognostic factors in patients who have undergone craniotomy for melanoma brain metastases (MBM) before 2011 at the University of Pittsburgh Medical Center (UPMC). We have investigated the prognostic or predictive role of these histopathologic factors in a more contemporary craniotomy cohort from the University of North Carolina at Chapel Hill (UNC-CH). We have also sought to understand better how various immune cell subsets, angiogenic factors, and blood vessels may be associated with clinical and radiographic features in MBM.

**Methods:**

Brain tumors from the UPMC and UNC-CH patient cohorts were (re)analyzed by standard histopathology, tumor tissue imaging, and gene expression profiling. Variables were associated with overall survival (OS) and radiographic features.

**Results:**

The patient subgroup with high TILs in craniotomy specimens and subsequent treatment with immune checkpoint inhibitors (ICIs, n=7) trended to have longer OS compared to the subgroup with high TILs and no treatment with ICIs (n=11, p=0.059). Bleeding was significantly associated with tumor volume before craniotomy, high melanoma-specific expression of basic fibroblast growth factor (bFGF), and high density of CD31+αSMA- blood vessels. Brain tumors with high versus low peritumoral edema before craniotomy had low (17%) versus high (41%) incidence of brisk TILs. Melanoma-specific expression of the vascular endothelial growth factor (VEGF) was comparable to VEGF expression by TILs and was not associated with any particular prognostic, radiographic, or histopathologic features. A gene signature associated with gamma delta (gd) T cells was significantly higher in intracranial than same-patient extracranial metastases and primary melanoma. However, gdT cell density in MBM was not prognostic.

**Conclusions:**

ICIs may provide greater clinical benefit in patients with brisk TILs in MBM. Intratumoral hemorrhage in brain metastases, a significant clinical problem, is not merely associated with tumor volume but also with underlying biology. bFGF may be an essential pathway to target. VEGF, a factor principally associated with peritumoral edema, is not only produced by melanoma cells but also by TILs. Therefore, suppressing low-grade peritumoral edema using corticosteroids may harm TIL function in 41% of cases. Ongoing clinical trials targeting VEGF in MBM may predict a lack of unfavorable impacts on TIL density and/or intratumoral hemorrhage.

## Introduction

Recent clinical trials in patients with active melanoma brain metastases (MBM) have shown that several systemic treatments that were initially approved for patients with metastatic melanoma (MM) without brain metastases also provide clinical benefit in distinct MBM patient subgroups (1–4). This clinical benefit has been partially explained by the high degree of biological similarity between brain metastases and extracranial metastases (EcM) (5, 6). Combination PD1 and CTLA4 blockade has a more durable clinical benefit than combination BRAF and MEK blockade in patients with active MBM (1–3).

Despite the advances in managing patients with MM, central nervous system metastases remain an ominous prognostic factor (7). It is not surprising that the clinical benefit from systemic treatments is more significant in patients with asymptomatic than symptomatic brain metastases (8). Asymptomatic patients with brain metastases are more frequently detected during routine screening for MM (9). Before the advent of targeted and immune checkpoint blockade therapies, patients with asymptomatic brain metastases lived longer than patients with symptoms from brain metastases (9). Symptomatic patients may have a high intracranial tumor burden with peritumoral edema and intratumoral hemorrhage. Brain metastases from melanoma have a higher propensity to hemorrhage than brain metastases from other solid tumors (10, 11), a complication with potentially fatal outcome.

Peritumoral edema in MBM positively correlates with tumor volume of brain metastatic melanomas, but not with a response to PD1 inhibitors, progression-free, or overall survival (OS) (12). Symptom control for patients with symptomatic MBM may involve craniotomy, radiation, and the use of corticosteroids or anti-epileptic agents. Corticosteroids may improve peritumoral edema, but are immunosuppressive and may reduce response to systemic treatments (13). Steroid-sparing treatments for peritumoral edema that target the vascular endothelial growth factor-A (VEGF-A) pathway are standard-of-care agents in progressive glioblastoma (14) and under clinical development in patients with MBM with concerns about safety and efficacy (clinicaltrials.gov NCT02681549).

We have previously shown in the University of Pittsburgh Medical Center (UPMC) craniotomy cohort that a higher density of tumor-infiltrating lymphocytes (TILs) and low hemorrhage were associated with longer OS following craniotomy (5). This study investigated whether any of these factors are also significant in a more contemporary independent craniotomy cohort. We extend our previously published gene expression profiling of patients with MBM from UPMC to report a more comprehensive analysis of the brain tumor microenvironment and its differences compared to that from same-patient EcM and primary melanoma (PrM). Finally, we have investigated the expression of several angiogenic cytokines, blood vessel density, and their potential association with hemorrhage and TIL density. By understanding the association among various radiographic, molecular, and histopathologic factors in patients with symptomatic MBM, we can identify predictors of response to existing systemic treatments and plan for the next generation of systemic treatments for this adverse prognosis group. Our results provide novel insights about brain tumor microenvironment-specific immune cell subsets, molecular, and radiographic parameters associated with intratumoral hemorrhage.

## Patients, Materials and Methods

### Patients and Clinical Information

Under an institutional review board-approved protocol, de-identified cases from patients who underwent craniotomy for MBM at the University of North Carolina at Chapel Hill (UNC-CH) were obtained by performing a CoPath pathology search followed by retrieval of formalin-fixed paraffin-embedded (FFPE) tumor blocks from craniotomy. Methodology for obtaining clinical information (demographics, melanoma characteristics, and local/systemic treatments before/after craniotomy), detailed histopathologic, and immunohistochemical (IHC) analysis for various immunologic and other markers have been previously described (5). Disease-specific OS was used as the sole clinical endpoint; patients with death other than melanoma were censored at the time of last follow-up.

We captured radiographic details about their metastatic brain disease before craniotomy from the brain magnetic resonance imaging (MRI) scan with intravenous (IV) contrast performed immediately before craniotomy, More specifically, we captured information regarding the degree of the peritumoral edema of the brain parenchymal lesion that was resected as ‘absent,’ ‘present/low,’ and ‘present/high.’ We calculated the tumor volume of the resected brain lesion by multiplying the length of each side and as reported in the neuropathology sign-out. We only calculated tumor volumes for cases whose tumor was resected in its entirety and not from stereotactic brain biopsies.

### Tumor Tissue Studies

#### Histopathology

We stained whole tissue sections of 5-µm thickness from available FFPE tumor blocks with hematoxylin and eosin (H&E). A board-certified neuropathologist (DGT) examined for tumor content and quality by light macroscopy review of H&E-stained sections from the UNC-CH melanoma craniotomy cohort.

#### Tumor Imaging Using Immunohistochemistry and Two-Color Immunofluorescence

##### Sources of Antibodies

We used the following primary antibodies for single-color IHC. Hypoxia-inducible factor-1 alpha (HIF1α, mouse monoclonal, clone 54, 610959, BD Biosciences, San Jose, CA; 1:100, 3-h incubation), CD31 (mouse monoclonal, clone JC70A, M0823, DAKO, Carpinteria, CA; 1:50, 1-h incubation), basic fibroblast growth factor (bFGF, mouse monoclonal, clone bFM-2, 05-118, Upstate/Millipore, Billerica, MA; 1:200, 1-h incubation), VEGF-A (rabbit polyclonal, sc-152, Santa Cruz Biotechnology, Santa Cruz, CA; 1:200, 1-h incubation), T-cell receptor delta chain (TCR δ-chain; mouse monoclonal, sc-100289, Santa Cruz; 1:400, overnight incubation).

We used the following primary antibodies for two-color immunofluorescence (IF). Angiopoietin-2 (Ang2, mouse monoclonal, clone MM0020-1F29, sc-101441, Santa Cruz Biotechnology; 1:40, 4-h incubation), alpha-smooth muscle actin (αSMA, mouse monoclonal, clone alpha sm-1, PA0943, Leica Microsystems, Inc., Buffalo Grove, IL; ready-to-use, 15-min incubation), CD31 (mouse monoclonal, clone JC70A, M0823, DAKO; 1:40, 3-h incubation).

##### Tissue Staining for Tumor Imaging

We carried single-color IHC and dual-color IF stains in the Leica Bond-III fully automated staining system (Leica Microsystems) at the Translational Pathology Laboratory, UNC-CH, as previously described (15–18) except for the TCR δ-chain and the Prussian Blue stain. Slides were dewaxed in Bond™ Dewax solution (AR9222, Leica Biosystems, Wetzlar, Germany) and hydrated in the Bond Wash solution (AR9590, Leica Biosystems). Epitope retrieval of HIF1α, CD31, and VEGF proteins was performed for 30 min at 100°C in Bond Epitope Retrieval Solution 1, pH 6.0 (AR9961, Leica Biosystems). We used the Bond Epitope Retrieval Solution 2, pH 9.0 (AR9640, Leica Biosystems) for antigen retrieval of bFGF (20 min). αSMA did not require epitope retrieval. After pretreatment, we incubated slides with primary antibodies described above for single-color IHC.

We performed the following two-color IF stains. First, the αSMA/CD31 double stain to differentiate and highlight vessel-like structures bearing endothelial cells (CD31+), which are associated with pericytes (αSMA+). Under this concept, “immature” vessels are αSMA-/CD31+ whereas “mature” vessels are αSMA+/CD31+) (19–21). Second, the Ang2/CD31 double stain to highlight areas with vascular remodeling and sprouting angiogenesis (22, 23).

We performed single chromogen detection by IHC using Bond Polymer Refine Red Detection (DS9390, Leica Biosystems) and dual-color IF detection using Bond Research Detection Kit (DS9455, Leica Biosystems) supplemented with Leica Novolink Polymer Detection System (RE7200, Leica Biosystems) and EnVision+ System-HRP, Labeled Polymer, anti-mouse (K4001, DAKO). We performed visualization using the PerkinElmer tyramide signal amplification (TSA)-Cy3 (SAT704A001EA, PerkinElmer, Inc., Waltham, MA) or TSA-Cy5 (SAT705A001EA5) kits. For nuclear counterstain, we used hematoxylin (Leica Microsystems) or the Hoechst 33258 dye (Life Technologies, Carlsbad, CA) for IHC and IF, respectively. We included positive and negative controls (no primary antibody) for each stain. We stained each of the tissue slides by IF, and we scanned them into Aperio FL. Following scanning, IF-stained tissue slides were subsequently stained with H&E to assist in better delineation of regions of interest (ROI; see “imaging analysis”).

The TCR δ-chain single-color IHC and Prussian blue stains were performed at the Histology Research Core Facility, UNC-CH. Regarding the TCR δ-chain single-color IHC stain, tissue slides were dried and then baked at 60°C for 90 min. After deparaffinization and hydration, heat-induced epitope retrieval was performed using HIER Buffer L (TA-135-HBL Thermo Fisher Scientific, Waltham, MA). Endogenous peroxidases were blocked using 3% hydrogen peroxide for 10 min at room temperature. Tissues were then blocked using 10% normal goat serum for 1 h at room temperature and incubated in the primary antibody. Following incubation with biotinylated goat anti-mouse IgG (115-065-166, 1:500 dilution; Jackson ImmunoResearch Laboratories, Inc, West Grove PA) for 1 h at room temperature, tissues were treated with ABC-HRP (PK-6100, Vector Laboratories, Burlingame, CA) and visualized using ImmPACT VIP peroxidase substrate (Vector, SK-4605). Finally, tissues were counterstained with 0.5% methyl green, dehydrated, cleared, and cover-slipped using the DPX mounting medium (13512, Electron Microscopy Sciences, Hatfield, PA). For the Prussian blue stain, tissue slides were dried and then baked at 60°C for 90 min. After deparaffinization and hydration, tissues were submerged in equal parts 20% hydrochloric acid and 10% potassium ferrocyanide for 40 min at room temperature. Tissues were washed in three changes of distilled water and counterstained with nuclear fast red for 5 min. Finally, tissues were rinsed in two changes of distilled water, dehydrated, cleared, and cover-slipped.

#### Tumor Tissue Studies Scoring

##### Manual (Pathologist)

Neuropathologist (DGT) scored degree of host response (i.e., immune infiltrate and gliosis, if stromal contexture was detectable), intratumoral hemorrhage, and necrosis by light macroscopy review of H&E-stained sections from the UNC-CH melanoma craniotomy cohort. We used the Prussian blue stain to differentiate out melanin from hemosiderin. We used the semiquantitative scale (0, 1+, 2+, 3+) for the H&E-based stains and the Prussian Blue stain as we have previously described (5). To quantify gamma delta T cells (γδT cells), a rare (5%) immune cell population (24), the neuropathologist measured the total number of anti-TCR δ H-41 antibody-positive cells seen in 10 high power fields (HPF) under 40X magnification. We used the following semiquantitative system; absent, few/rare (<5 cells), moderate (5–10 cells), many (>10 cells).

##### Semi-Automated (Computer-Assisted) Scoring-Tumor Imaging Analysis

We performed imaging analysis for the HIF1α, VEGF-A, bFGF, and CD31 single-color IHC stains and the two-color IF stains in the Translational Pathology Laboratory, UNC-CH, as previously described (15, 17, 18, 25). We obtained digital slides by scanning with a 20X objective IF-stained slides using the Aperio FL Slide Scanner in the DAPI (Hoechst 33258; H3569, Invitrogen, Carlsbad CA), Cy3-green (αSMA, Ang2), and Cy5-red (CD31) channel. Tissue slides that were stained with IF and then scanned into Aperio FL were subsequently stained with H&E stain and scanned into Aperio Scanscope XT to assist in detecting different tissue compartments within the brain. For the IHC-stained slides, we used the Aperio Scanscope XT Slide Scanner (Leica Biosystems).

Using a digital pen (ePathology ImageScope software, version 11.2.0.780; Leica Biosystems) on the H&E-stained section of the previously stained slide (IHC) or the same slide (two-color IF), the neuropathologist defined the following four tissue “compartments” within MBM: melanoma cell predominant/rich, reactive glia, normal brain, and compartment enriched for lymphocytic clusters. We excluded areas of necrosis, hemorrhage, and pigmentation from the analysis. We used the Genie histology pattern recognition tool (version 7.1.100.1248, Leica Biosystems) to develop a classifier that separates tissue from non-tissue areas (e.g., glass) or other artifacts (chromogen precipitation, tissue section folds on the slide) for further analysis. We used the Color Deconvolution v9 area quantification algorithm (ImageScope) software to calculate the average H-score (0-300) of the VEGF, bFGF, and HIF1α stain for each of the four brain tumor tissue compartments.

For analysis of two-color IF stains and the blood vessel density of tissue slides stained by single-color IHC using an antibody against CD31, we used the Definiens Architect XD 2.1.1 with Tissue Studio (version 3.6.1; Definiens Inc., Cambridge, MA). For density analysis of CD31+ blood vessels that are surrounded by pericytes (αSMA+), or not, or undergo remodeling/sprouting (Ang2+), or not, we selected ROIs based on αSMA and Ang2 signal detection following tissue and background separation. We trained the Definiens Composer algorithm to detect positive and negative ROIs. Using the Vessel Detection algorithm of the Definiens Tissue Studio, we detected CD31+ blood vessels in αSMA-negative and Ang2-positive/negative ROIs. The following vessel stain detection parameters were applied: we set the intensity threshold to 3,500 (for FL images having 16-bit image depth); minimum and maximum stain vessel area to 50 µm (2) and 500,000 µm (2), respectively (which corresponds to a blood vessel with a diameter approximately 8 µm and 800 µm, respectively); the gap to close to 5 µm.

### Data Analysis

#### Statistical Analysis for Clinical Data and Tumor Tissue Imaging Studies

Descriptive statistics were used to summarize the average H-score for each covariate analyzed by IHC (HIF1α, VEGF, bFGF) and expressed in melanoma versus each of the other non-melanoma compartments (reactive glia, normal brain, immune clusters) of the brain tumor microenvironment. We performed Kruskal-Wallis test to assess whether there was any significant difference in each covariate expression in different brain tumor compartments. If results were statistically significant (p<0.05), we performed a Mann-Whitney test to assess any significant difference in the H-score between melanoma and each of the other non-melanoma compartments. We generated analysis and graphs using GraphPad Prism version 8 (GraphPad Software, San Diego, CA).

Generalized estimating equations (GEE) were used to explore the association between the H-score (or blood vessel density) of each covariate assessed in the melanoma compartment and TIL density or intratumoral hemorrhage (26). In cases where GEE did not reliably fit, we used regression models instead. Given the lack of knowledge about the cause-and-effect relationship between the expression (or blood vessel density) and TIL/intratumoral hemorrhage, these models were run twice for each variable, treating H-score or blood vessel density as the outcome and TIL/intratumoral hemorrhage as the covariate, and vice versa. We performed the analysis using R (r-project.org). We calculated the Kendall τ rank correlation to explore the association among histopathologic covariates that were semi-quantitatively scored (0, 1+, 2+, 3+), peritumoral edema (0, present/low, present/high), and tumor volume of the resected craniotomy specimen.

We used the Kaplan-Meier method for survival analysis to assess the prognostic significance of the five histopathologic covariates that were semi-quantitatively scored and dichotomized by convention as “high” (2+, 3+) and “low/absent” (0, 1+); i.e., TILs, hemorrhage, gliosis, necrosis, and pigmentation, as we have previously described (5). For γδT cells, we dichotomized results as present or absent. We performed analysis and generated graphs using GraphPad Prism version 8 (GraphPad Software). For survival analysis of continuous covariates assessed by computer imaging analysis using either IHC or IF, Cox proportional hazards models with robust standard errors (to account for two observations for some patients) were used. We performed additional exploratory survival analysis using the optimal cut-point method for “high” vs. “low” expression for covariates assessed by computer imaging. The clinical question was whether patients who lived longer demonstrated “higher” or “lower” expression of the particular biomarker/covariate for a given cut-point. *P*-values lower or equal to 0.05 were considered significant. We reported unadjusted *p*-values given the exploratory nature of the optimal cut-point analysis. We performed the analysis using R (r-project.org).

### Bioinformatics Analysis of Immune and Glial Subtype of Same-Patient Primary Melanoma, Extracranial Metastases, and Brain Metastases (UPMC Craniotomy Cohort Only)

Methods corresponding to the gene expression profiling of RNA extracted from UPMC craniotomy samples have been reported elsewhere (5). Briefly, melanoma tissue was macrodissected from consecutive FFPE, adjacent 5µM-thick tissue sections using an adjacent H&E-stained tissue section as a guide to discriminate viable tumor from non-necrotic tumor and normal tissue. Tissue pellets of interest were deparaffinized and RNA was purified and incubated with the Human Ref-8, version 3, BeadChips (Illumina, San Diego, CA). Quintile-normalized gene expression profiling of samples corresponding to craniotomies at UPMC (5) was annotated using reference files (https://support.illumina.com/array/downloads.html). A normalized gene expression matrix was developed using the ‘affy’ package (https://www.bioconductor.org/packages/release/bioc/html/affy.html). We calculated a normalized gene expression score for each tumor sample after the mean expression of genes comprising each gene signature. Scores were then centered by subtracting the mean across all tumor samples. We subsequently used computed scores for four downstream analyses. First, to assess whether melanomas metastatic to lymph nodes had a higher degree of distinct immune cell subtypes compared with melanomas metastatic to non-lymph nodes (e.g., lung, liver) merely due to possible “contamination” by adjacent normal lymphoid tissue remnants. We therefore compared various immune cell gene signatures (27) between EcM in lymph nodes vs. EcM in non-lymph node metastases using the Wilcoxon rank sum test. Second, we performed comparisons of tumor types (i.e., PrM, EcM, and MBM) for various immune cell gene signatures across all specimens (unpaired) using the Kruskal-Wallis test across all three groups, followed by a Wilcoxon rank sum test between each tumor type (EcM vs. MBM, EcM vs. PrM, and MBM vs. PrM) and gene signature. Third, we performed a separate pairwise analysis for same-patient MBM vs. EcM tumor specimens using the Wilcoxon paired test. Fourth, we investigated the association between gene expression signatures corresponding to the MBM samples and OS, defined as the time from craniotomy to death from melanoma, using Pearson’s correlation test. We conducted survival analysis using a univariate Cox proportional hazards regression model with each gene signature being a covariate. We adjusted *p*-values for multiple testing within comparison groups (EcM vs. MBM, EcM vs. PrM, and MBM vs. PrM) using the Benjamini-Hochberg procedure. All nominally significant *p-*values were considered significant at *p* < 0.05.

The majority of gene signatures used for our analyses were previously published immune gene signatures (27). To better understand the prognostic significance of the resident cell population in MBM, we developed and calculated gene signatures specific for astrocytes, oligodendrocytes, neurons, and microglia. For the three non-microglial populations, we performed extensive literature search filtering out genes that were previously listed as specific for each of the three non-microglial populations in the mouse brain (27) based on two criteria. First, genes must have a relevant function for the human brain and not for other human organs. Second, they should not be differentially expressed in cutaneous melanoma. Differential expression was defined by convention as differentially expressed (Z-score greater than 2.0) genes in less than 3% of the tumor specimens from the Cancer Genome Atlas Project in Cutaneous Melanoma (www.cbioportal.org) (28). We performed a similar literature search for the microglia population to filter out genes listed in a previous report on gene expression profiling of mouse brain microglia (29). The remaining genes must be related to human brain function and lack expression in cutaneous melanoma. **Supplementary Figure 1** describes our filtering strategy and individual genes that comprise each signature corresponding to the resident brain cells.

## Results

### Clinicopathologic and Molecular Characteristics of the University of North Carolina Melanoma Craniotomy Cohort

Sixty-two patients with MBM were treated at UNC-CH and underwent craniotomy between March 1992 and February 2020. Thirty-three of these patients underwent craniotomy after 2011. **Table 1** shows the clinicopathologic characteristics of the UNC-CH cohort. Like the UPMC craniotomy cohort, a high percentage of patients had melanoma of unknown primary (26%). Of the 39 patients whose melanoma was tested for *BRAFV600E* mutations using various assays (molecular and IHC), 14 (36%) were positive, whereas 7 of the 24 (29%) patients whose tumor was also tested for *NRASQ61* codon mutations were positive. Although only seven patients’ tumors were tested with more extensive targeted sequencing panels (FoundationOne and UNCseq) (30), four had TERT promoter mutations, and five had splice-site or stop-codon mutations in the *NF1* gene. Before craniotomy, seven (11%) patients had received radiation to the resected brain tumor, 17 (27%) patients received immunotherapy, and three (5%) patients received targeted therapies (vemurafenib or imatinib). Following a craniotomy, 21 patients (34%) received ipilimumab and/or PD1 inhibitors, and twelve patients (19%) received BRAF and/or MEK inhibitors. At a median follow-up of 9.9 months (range 0.1 to 190.8 months), 51 patients (82.2%) had died from MM.

**Table 1:** Clinicopathologic characteristics of the University of North Carolina at Chapel Hill melanoma craniotomy cohort (N=62).

**Sex**				
	Male			41
	Female			21
**Age** at Craniotomy (median), range (years)				55 (30,85)
**Race**				
	Caucasian			59
	Hispanic/Latino			2
	African American			1
**Melanoma Subtype**				
	Cutaneous			45
	Head and Neck			11
	Trunk			18
	Extremity			19
	Multiple primaries			2
	Unknown			5
	Unknown			16
	Mucosal			1
**Molecular Characteristics**				
	Non-tested			23
	Tested			39
	Immunohistochemistry			5
		*BRAFV600E*-mutant		3
	Pyrosequencing			15
		*BRAFV600* mutation (E/K, out of 15 tested)		5 (4/1)
		*NRASQ61* mutation (out of 5 tested)		2
		*CKIT* mutation (out of 5 tested)		1
	Illumina 26-gene panel			12
		*BRAFV600* mutation (E/K)		6 (5/1)
		*NRASQ61* mutation		3
		*PTEN* mutation		3
		*TP53*		1
	FoundationOne			6
		*BRAFV60*0		0
		Non-BRAFV600		2
		*NRASQ61* mutation		2
		TERT promoter mutations		4
		*CDKN2A* mutation/deletion		5
		*NF1* splice site/stop codon mutations		4
		Other (*PTEN*, *EZH2*, *SETD2*, *RAC1*)		4
	UNCseq			1
		*BRAFV600*		0
		*NF1* splice site/stop codon mutations		1
**Craniotomy Tumor Specimen Volume (cm^3^, median, range)**			3.97 (0.06, 124.2)	
**Peritumoral Vasogenic Edema of the Resected Lesion (brain scan)**				
	Absent			5
	Present, low			32
	Present, high			23
	Not reported			2
**Brain Radiation Prior to Craniotomy**				
	No			55
	Yes			7
**Systemic Treatments Prior to Craniotomy**				
	No			36
	Yes			26
	Immunotherapies			17
		Adjuvant		14
		High-dose interferon		11
		Immune checkpoint inhibitors		3
		For stage IV		5
	Chemotherapies			6
	Targeted Treatments			3
**Systemic Treatment After Craniotomy**				
	No			27
	Yes			35
	Immunotherapies			24
		PD1 inhibitors only		7
		CTLA4 inhibitors only		6
		PD1 and CTLA4 inhibitors		8
		Other (high-dose bolus IL-2, GM-CSF)		3
	Chemotherapies			12
	Targeted Treatments			12
	Unknown			1

### Association of Histopathologic Characteristics of Melanoma Brain Metastases With Molecular Features, Radiographic Parameters, and Clinical Follow-Up in the UNC-CH Melanoma Craniotomy Cohort

**Figure 1:**
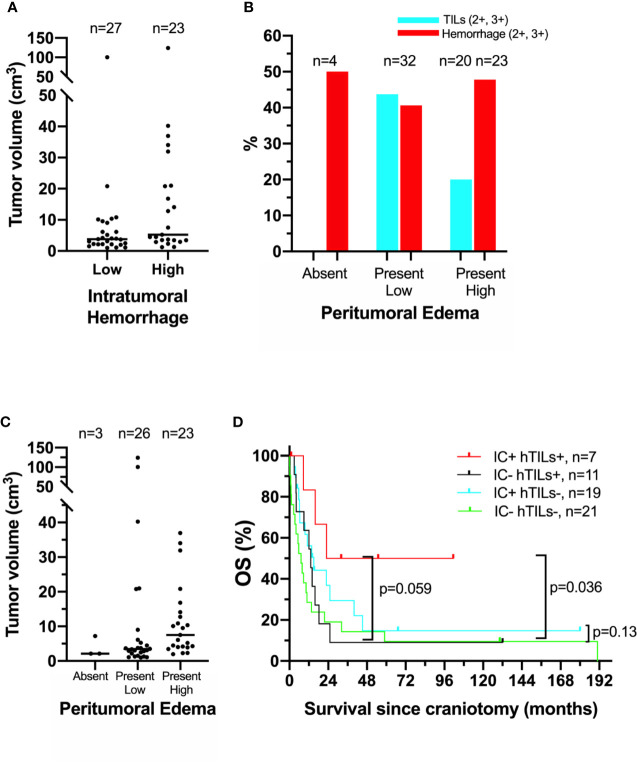
Association of clinical, histopathologic, and radiographic features in the University of North Carolina at Chapel Hill melanoma craniotomy cohort. See methods for details: **(A)** individual tumor volumes with high (2+, 3+) versus low/absent (0, 1+) intratumoral hemorrhage, as measured by histopathologic analysis on hematoxylin and eosin (H&E)-stained representative tissue sections. **(B)** Incidence of high (2+, 3+) tumor-infiltrating lymphocytes (hTILs) and intratumoral hemorrhage, as measured by histopathologic analysis on H&E--stained tissue sections, in relation to the degree of peritumoral edema, as measured by brain magnetic resonance imaging. **(C)** individual tumor volumes in brain tumors with absent, present/low, and present/high peritumoral edema. **(D)** Exploratory survival analysis (Kaplan-Meier method) according to the presence of hTILs, as measured by histopathologic analysis on H&E-stained tissue sections and treatment with immune checkpoint inhibitors (IC) following craniotomy.

Our previous histopathologic analysis of MBM tissues from the UPMC craniotomy cohort had shown that a high density of TILs and low intratumoral hemorrhage were associated with favorable prognosis (5). However, in the UNC-CH craniotomy cohort neither high density of TILs nor low intratumoral hemorrhage were significantly associated with prolonged OS (**Table 2**). Intratumoral hemorrhage was the only histopathologic feature significantly correlated with the volume of tumor specimens resected during craniotomy (Kendall rank τ=+0.30, *p* <0.01, **Figure 1A**). None of the five histopathologic features correlated with the degree of peritumoral edema detected in the radiographic imaging study immediately before craniotomy significantly. However, all four tumors with histopathologic evidence of low/absent TILs (0,1+) had no radiographic evidence of peritumoral edema. In contrast, 41% (13/32) of patients with present/low peritumoral edema by brain MRI scans had tumors bearing brisk TILs (2+, 3+), and only 17% (4/23) tumor with high peritumoral edema had tumors with brisk TILs (**Figure 1B**). Of all the histopathologic and radiographic features, only the volume of the tumor specimen resected during craniotomy significantly correlated with the degree of peritumoral edema (Kendall rank τ=+0.32, p<0.01, **Figure 1C**). The median OS of patients who had high TILs in their craniotomy specimens and received immune checkpoint inhibitors following craniotomy trended to be longer compared to that of patients who had high TILs but did not receive immune checkpoint inhibitors following craniotomy (62.2 vs. 13.2 months, log-rank p=0.059). Immune checkpoint inhibitors following craniotomy did not significantly prolong OS in patients who had low/absent TILs in their craniotomy specimens (median OS was 15 vs. 7.2 months, log-rank p=0.13, **Figure 1D**).

**Table 2:** Histopathologic factors in relation to overall survival (OS) in patients with melanoma brain metastases from the UNC-CH craniotomy cohort.

Variable	No. of Patients	Median OS (months)	Log-Rank *P*	Kaplan-Meier-Estimator HR* (95% CI)
*Immune Infiltrate*				
Low/absent	41	9.5	0.23	1.46 (0.80–2.65)
High	18	15.7		
Non-assessable	3			
*Hemorrhage*				
Low/absent	33	13.6	0.14	0.66 (0.36–1.19)
High	26	8.7		
Non-assessable	3			
*Gliosis*				
Low/absent	24	13.4	0.25	0.66 (0.31–1.43)
High	15	6.8		
Non-assessable	23			
*Necrosis*				
Low/absent	46	12.0	0.44	0.77 (0.37–1.60)
High	13	9.5		
Non-assessable	3			
*Melanin (Prussian blue negative pigment)*				
Low/absent	38	10.9	0.61	0.86 (0.45–1.6)
High	17	8.6		
Non-assessable	7			

Each variable was assessed semiquantitatively (0, 1+, 2+, 3+) and was dichotomized to low/absent (0, 1+) and high (2+, 3+). See methods. HR, hazard ratio; CI, confidence interval.

### Prognostic Significance of Immune and Glial Subtype Gene Expression Signatures in Melanoma Brain Metastases From the UPMC Craniotomy Cohort

Gene expression profiling analyses of MBM, published by us and others, have consistently shown that effector T cells are associated with prolonged OS (5, 31). To comprehensively characterize immune cell subtypes that may have prognostic significance in MBM, we re-analyzed our previously published gene expression profiling data that were available from a subset (N=29) of the previously published UPMC craniotomy cohort (5). The clinicopathologic characteristics corresponding to these 29 specimens are shown in **Table 3**. No patients’ resected brain tumors had received radiation therapy before craniotomy. Only three patients received immunotherapies following craniotomy. Of the extracranial tumors, more than half were derived from melanoma-infiltrated lymph nodes. We then calculated gene expression scores for various previously published immune cell types (27) and novel gene expression signatures that correspond to neurons and the three most abundant glial types within the central nervous system, namely astrocytes, oligodendrocytes, and microglia for this UPMC cohort. **Supplementary Figure 2** shows gene expression scores corresponding to 37 different gene expression signatures for the 29 melanoma craniotomy specimens with OS. Survival analysis using univariate Cox proportional hazards regression models showed four signatures (*Cytotoxic_cells*, *NK_CD56dim_Cells*, *T_Cells*, *LCK*) that were significantly associated with OS (**Supplementary Figure 3**). However, none of these associations remained significant after correction for multiple comparison testing. Our analysis confirmed prior reports about the prognostic significance of effector T cells and natural killer cells in MBM but failed to reveal that other immune cell subsets or resident brain cell types may have prognostic significance.

**Table 3:** Clinicopathologic characteristics of patients from the University of Pittsburgh Medical Center craniotomy cohort (n=32).

**Sex**			
	Male		23
	Female		9
**Age at Craniotomy (years), median (range)**			55 (21,83)
**Median Follow-up (days), median (range)**			215.5 (18,1766)
**Status at last follow-up**			
	Alive		5
	Deceased		27
**Metastatic organ of origin for extracranial tumors**			
	Lymph nodes		15
	Non-lymph nodes		13
**Brain Radiation to the Resected Brain Mass Prior to Craniotomy**			
	No		32
	Yes		0
**Systemic Treatments Prior to Craniotomy**			
	No		14
	Yes		18
		Immunotherapy only	13
		Chemotherapy	3
		Immunotherapy and Chemotherapy	2
**Brain Radiation After Craniotomy**			
	No		10
	Yes		22
**Systemic Treatment After Craniotomy**			
	No		19
	Yes		13
		Immunotherapy only	1
		Chemotherapy only	10
		Immunotherapy and Chemotherapy	1
		Immunotherapy and Targeted Therapy	1

Please note that craniotomy specimens from three patients did not undergo gene expression profiling, although the primary melanoma and extracranial metastases did. See **Supplementary Figure 1** from *Hamilton et al. Cancer 2013, PMID 23695963*, for details.

**Figure 2:**
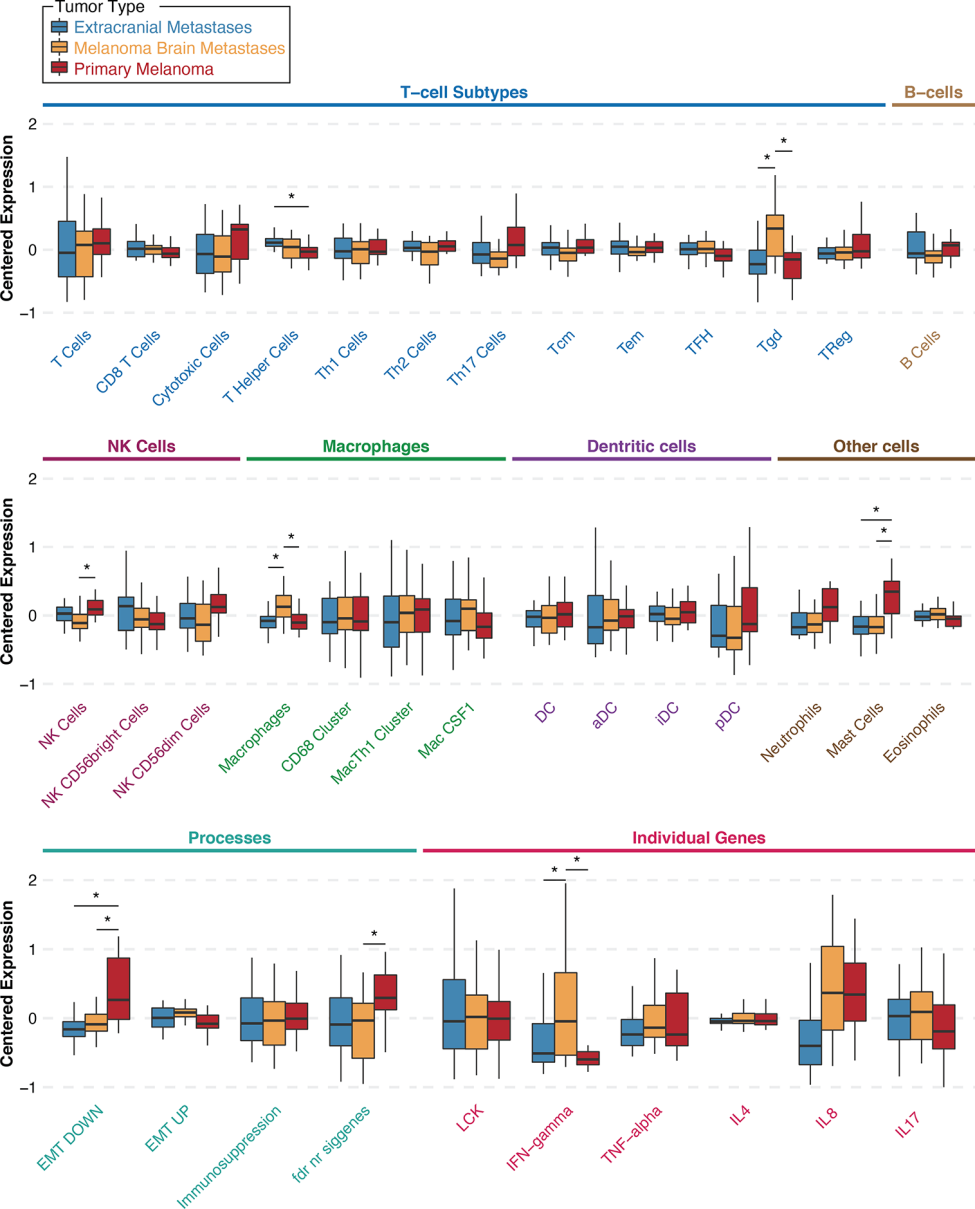
Gene signature scores for extracranial metastases (*blue*), melanoma brain metastases (*orange*), and primary melanoma (*red*) samples from the University of Pittsburgh Medical Center craniotomy cohort. Results are shown as boxplots with median and 95% confidence intervals for each signature and tumor type. Asterisks indicate two-way comparisons that are statistically significant at a nominal *p*-value <0.05. Abbreviations: Th, T helper; Tcm, central memory T cells; Tem, effector memory T cells; TFH, follicular helper T cells; Tgd, γδT cells; TReg, regulatory T cells; NK, natural killer cells; Mac, macrophages; DC, dendritic cells; aDC, activated DCs; iDC, immature DCs; pDC, plasmacytoid DCs; EMT, epithelial-to-mesenchymal transition; LCK, lymphocyte-specific protein tyrosine kinase; IFN, interferon; TNF, tumor-necrosis factor; IL, interleukin.

To investigate whether specific immune cell populations are differentially present in MBM than same-patient EcM and same-patient PrM, we re-analyzed gene expression data from our previously published UPMC melanoma craniotomy cohort (n=72) for 33 different gene expression signatures. First, to assess whether EcM in lymph nodes (n=13) had differences in distinct tumor-infiltrating immune cell subsets compared with EcM in non-lymph nodes (n=15), we compared gene signatures corresponding to 27 different immune cell subsets (27). Only the gene signature corresponding to *B_Cells* was significantly higher in EcM in lymph nodes versus EcM in non-lymph nodes (Wilcoxon rank sum test, p=0.011, **Supplementary Figure 4**). **Figure 2** shows gene expression scores for each immune cell subtype signature according to tumor type and across all specimens (unpaired analysis; 29 MBM, 28 EcM, and 15 PrM). As anticipated in metastatic samples, the *EMT_DOWN* gene expression signature score was significantly lower in both EcM and MBM compared to PrM. The gene expression signature score corresponding to *MastCells* was also significantly lower in both metastatic specimen types compared with PrM (Wilcoxon rank sum test, *p*=0.033 for the EcM comparison and p=0.036 for the MBM comparison). The gene expression signature scores corresponding to *macrophages* (**Supplementary Table 1**) and *gamma delta T cells* (γδT cells, Tgd) were significantly higher in MBM compared with same-patient EcM (*p*=0.033 and *p*=0.033, respectively) and PrM (*p*=0.036 and *p*=0.036, respectively). In contrast, the gene expression signature score corresponding to *NK Cells* was significantly lower in MBM compared with PrM (*p*=0.041), but not between MBM and EcM (*p*=0.275). Our more robust analysis on same-patient MBM and EcM (26 pairs) across various immune cell populations showed that MBM have significantly higher scores in gene signatures corresponding to *Tgd cells* and *macrophages* (Wilcoxon paired test, p-value 0.001 and 0.02, respectively) and significantly lower score in *central memory T cells* (Tcm, p=0.049). Our results suggest that the melanoma brain microenvironment is enriched with unique immune cell subsets compared to EcM and PrM.

**Figure 3:**
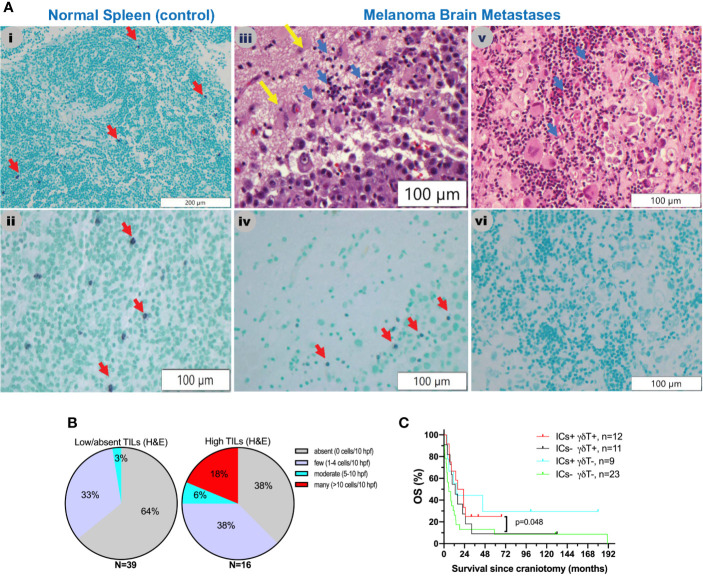
γδT cells in melanoma brain metastases from the University of North Carolina at Chapel Hill craniotomy cohort using a T-cell receptor (TCR) delta chain mouse monoclonal antibody. **(A)** Abundance of γδT cells in relation to tumor-infiltrating lymphocytes (TILs) as detected by routine hematoxylin and eosin (H&E) staining. See *Patients, Materials and Methods* for details. i and ii. Staining of a normal human spleen as a positive control. Please note that the γδT stain is violet (purple) membranous stain (red arrows), whereas the nuclear counterstain is light green. iii and iv. Representative images from a MBM that contains TILs (blue arrows, iii) and γδT cells (red arrows, iv). Yellow arrows indicate normal brain. v and vi. Representative images from a MBM containing TILs (blue arrows, v) but not γδT cells (vi). **(B)** Abundance of γδT cells in melanoma brain specimens that bear absent/low (left pie chart) versus high (2+, 3+) TILs (right pie chart). **(C)** Exploratory overall survival (OS) analysis (Kaplan-Meier method) according to the presence of TILs (H&E stain) and treatment with immune checkpoint inhibitors IICs following craniotomy.

Little is known about the presence and role of γδT cells in metastatic cancers to the brain. Driven by our gene expression profiling analyses, which showed that γδT cells are significantly higher in MBM than EcM, we sought to validate their presence and distribution in MBM. **Figure 3A** shows representative images of γδT cells in normal human spleen and MBM from the UNC-CH cohort. Of the 55 MBM that were analyzed for γδT cell abundance in the UNC-CH craniotomy cohort, 30 tumor specimens had no γδT cells, 19 had few γδT cells, and only six tumors had a moderate or high number of γδT cells. Of the 37 tumor specimens with present TILs by histopathologic analysis (1+, 2+, 3+), only 23 tumor specimens had present γδT cells **(** **Figure 3B** **)**. We explored gene expression of four cytokines produced by tumor-infiltrating immune cells, including γδT cells, given that tumor-infiltrating γδT cells can have opposing effects in cancer immunity according to the cytokine profile that they produce (32). **Figure 2** shows that of the four cytokines, IFNγ expression was significantly higher in MBM compared with PrM and EcM. Although these cytokines can be expressed by tumor-infiltrating immune cells other than γδT cells, our results raise the possibility that antitumor and pro-inflammatory cytokines are present in MBM. Status of γδT cells (present, absent) did not significantly influence OS of patients who received immune checkpoint inhibitors following craniotomy. Nevertheless, the OS of patients who had γδT cells in MBM and received immune checkpoint inhibitors following craniotomy was significantly longer compared to that of patients who neither had γδT cells in MBM nor received immune checkpoint inhibitors following craniotomy (19.35 vs. 5.7 months, log-rank p=0.048, **Figure 3C**).

#### Expression of HIF1α and Various Angiogenic Cytokines by Stromal and Melanoma Cells in the UPMC and UNC-CH Melanoma Craniotomy Cohort

**Figure 4:**
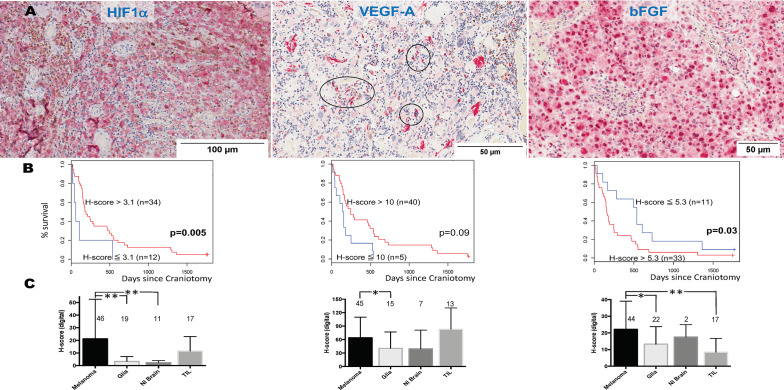
Expression of hypoxia-inducible factor-1 alpha (HIF1α), vascular endothelial growth factor A (VEGF-A), and basic fibroblast growth factor (bFGF) in melanoma brain metastases (MBM) from the University of North Carolina at Chapel Hill craniotomy cohort. **(A)** Representative tissue sections stained with immunohistochemistry. See *Patients, Materials and Methods* for details. Black circles indicate immune cell clusters. **(B)** Overall survival of patients with MBM according to the expression of HIF1α, VEGF-A, bFGF, and using optimal cut-point analysis. See *Patients, Materials and Methods* for details. **(C)** Expression (digital H-score) of HIF1α, VEGF-A, and bFGF within each of the four different compartments of MBM. The numbers above boxplots indicate the number of specimens for each measured variable. See *Patients, Materials and Methods* for details in the analysis. *p= 0.01-0.05; **p < 0.01.

We and others have previously shown that HIF1**α** expression is significantly higher in MM than primary melanomas and nevi (33). HIF1α directly regulates VEGF, the target for bevacizumab. Bevacizumab is currently being tested in combination with PD1 inhibitors in clinical trials for symptomatic MBM (clinicaltrials.gov NCT02681549). However, melanoma cells express other angiogenic cytokines independent from hypoxia signals, such as bFGF (34). VEGF, bFGF, and other angiogenic factors have been associated with angiogenesis and vasogenic edema in gliomas (35). To identify the pattern of expression of HIF1α and the two chief angiogenic cytokines in MBM, VEGF and bFGF, we stained representative tissue sections from craniotomy specimens corresponding to the UPMC cohort using single-color IHC (HIF1α, VEGF, bFGF, **Figure 4A**). We then performed imaging analysis by quantifying each protein’s expression in the four different cellular compartments of the tumor: melanoma, normal brain, reactive glia, and lymphocytic clusters. Overall, the expression (digital H-score) of all three proteins by melanoma cells was low-to-intermediate. Cox proportional hazards models for HIF1α (n=46), VEGF (n=45), and bFGF (n=46) expression in the melanoma compartment alone (digital H-score) showed that none was prognostic. More specifically, the hazard ratio for a 10-unit increase in H-score was 0.96 for HIF1α (*p*=0.24), 0.95 for VEGF (*p*=0.11), and 1.15 for bFGF (*p*=0.17). We performed an exploratory analysis using the survival information to pick the ‘optimal’ cut-point between “high” versus “low” expression of HIF1α, VEGF, and bFGF. As shown in **Figure 4B**, higher expression of HIF1α, and lower expression of bFGF in the melanoma compartment are associated with longer OS. Our results indicate that melanoma-specific VEGF expression may not have prognostic significance in MBM.

We then compared the protein expression (digital H-score) of HIF1α, VEGF, and bFGF among the four cellular compartments within MBM tumor samples. As shown in **Figure 4C**, there were differences in each protein expression among different cellular compartments. Although expression of HIF1α and bFGF was significantly higher in the melanoma compartment compared to other melanoma compartments, no such significant differences were seen with VEGF expression. In particular, VEGF expression by melanoma cells was not significantly different from that by TILs.

There are theoretical concerns that targeting VEGF in MBM may significantly increase intratumoral hemorrhage, but may favorably affect immune infiltration. We investigated the association between digital H-score of HIF1α, VEGF, and bFGF with hemorrhage and TILs. Using GEE models, or regression models when GEE could not be reliably fit, only bFGF digital H-score from the melanoma compartment was significantly positively associated with intratumoral hemorrhage (coeff = +5.74, *p*=0.02, **Figure 4A**, yellow arrows) and significantly negatively associated with TILs (coeff = -5.70, *p*=0.009). In contrast, VEGF digital H-score from the melanoma compartment tended to correlate negatively with hemorrhage (coeff = -9.82, *p*=0.08). We summarize the results from the associations with hemorrhage and TIL density in **Table 3**.

#### Blood Vessel Density in Melanoma, Reactive Glial, and Normal Brain Compartments in Brain Tumors From the UNC-CH Craniotomy Cohort

**Figure 5:**
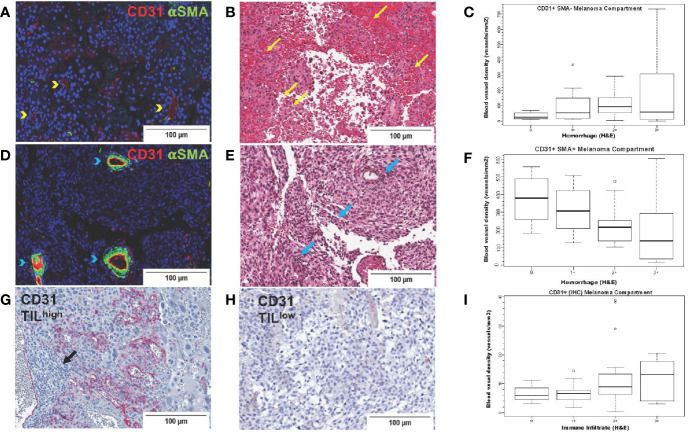
CD31 blood vessel density in melanoma brain metastases from the University of North Carolina at Chapel Hill craniotomy cohort. Images in the upper row **(A–C)** correspond to immature blood vessels (CD31+αSMA-); middle row images **(D–F)** correspond to mature blood vessels (CD31+αSMA-); lower row images **(G–I)** correspond to total (CD31+) blood vessels. Digital images that correspond to representative tissue sections stained with antibodies against CD31 [pseudocolored with red, **(A, D)**] and αSMA [pseudocolored with green, **(A, D)**] by two-color immunofluorescence (IF) and by CD31 by single-color immunohistochemistry **(G, H)** are shown. Tissue slides stained by two-color IF were subsequently stained with hematoxylin and eosin (H&E) **(B, E)**. Areas with CD31+αSMA- blood vessels [yellow arrowheads, **(A)**] trend (*p*=0.07) to have high intratumoral hemorrhage [yellow arrows, **(B)**], whereas areas with CD31+αSMA+ blood vessels [blue arrowheads, **(D)**] have significantly low/absent intratumoral hemorrhage [blue arrows, center image, **(E)**]. Areas with high [black arrows, **(G)**] versus low density of tumor-infiltrating lymphocytes (TILs) **(H)** are also shown. Boxplots showing correlation between blood vessel density of immature [CD31+αSMA-, **(C)**] and mature [CD31+αSMA+, **(F)**] blood vessels with hemorrhage. **(I)** shows density of CD31+ blood vessels with TILs (H&E).

To systematically investigate the prognostic significance of various types of blood vessels according to pericyte coverage and vessel sprouting/stability using unbiased computer imaging methods in craniotomy melanoma specimens, we measured total (CD31+, single-color IHC, n=43), mature (CD31+αSMA+, two-color IF, n=41), immature (CD31+αSMA-, two-color IF, n=41), stable/non-sprouting (CD31+Ang2+, two-color IF, n=29), and unstable/sprouting (CD31+/Ang2-, two-color IF, n=31) blood vessel density within the melanoma compartment. We did not find any significant differences in OS for patients bearing MBM according to blood vessel density of mature, immature, and stable/non-sprouting (CD31+Ang2+) blood vessels. The only exception was unstable/sprouting (CD31+Ang-) blood vessels; in other words, higher unstable/sprouting blood vessel density trended to be associated with favorable prognosis (HR=0.98 for a 10-unit increase in blood vessel density, *p*=0.08). Furthermore, the density of immature (CD31+αSMA-) blood vessels trended to correlate with hemorrhage positively (coeff = +43.17, *p*=0.07, **Figure 5A**), whereas the density of mature blood vessels significantly correlated with hemorrhage negatively (coeff =-61.44, *p*=0.01, **Figure 5B**). Furthermore, CD31+ blood vessel density significantly positively correlated with TIL density, irrespective of pericyte coverage (CD31+ coeff = 2.39, *p*=0.004; CD31+αSMA- coeff =51.08, *p*=0.03; CD31+αSMA+ coeff = 55.41, *p*=0.02, **Figure 5C**). We summarize the results from the associations with hemorrhage and TIL density in **Table 4**.

**Table 4:** Correlation between various parameters within the melanoma compartment and intratumoral hemorrhage or tumor infiltrated lymphocytic density in melanoma brain metastases (University of Pittsburgh Medical Center cohort).

Stain	Method	N	Association with Hemorrhage			Association with Immune Infiltrate		
			Model type	coeff	P-value	Model Type	coeff	P-value
HIF1α	IHC	46	GEE	−4.49	0.24	GEE	−7.80	0.06
VEGF	IHC	45	GEE	−9.82	0.08	RM	+0.87	0.90
bFGF	IHC	44	RM	+5.74	0.02	GEE	−5.70	0.009
CD31	IHC	43	GEE	+0.22	0.89	GEE	+2.39	0.004
CD31+αSMA-	IF	40	RM	+43.17	0.07	RM	+51.08	0.03
CD31+αSMA+	IF	40	RM	−61.44	0.01	RM	+55.41	0.02
CD31+Ang2-	IF	30	GEE	−34.92	0.26	GEE	+53.57	0.16
CD31+Ang2+	IF	29	GEE	−30.39	0.19	GEE	+3.77	0.89

Generalized estimating equation models (GEE) were used to account for multiple observations for some patients. Regression models (RM) were used when GEE could not be reliably fit. Digital H-score is the outcome, and hemorrhage is the covariate.

## Discussion

Inexpensive (H&E-stained sections) and reproducible histopathologic parameters can be useful as prognostic or predictive markers of response to systemic treatments for MM and can be potentially incorporated into standard neuropathology signoffs that only focus on diagnosis. We have previously reported that the density of TILs as assessed in H&E-stained sections and specific IHC stains against various T cell subsets (CD8) have prognostic significance in patients with brain metastases from melanoma and breast cancer (5, 36).

One of our most compelling findings in this study is the striking difference in the incidence of inflamed (i.e., high TILs) melanoma craniotomy specimens between the UNC-CH and UPMC cohorts (29% vs. 56%, respectively). The percentage of patients who received immunotherapies before craniotomy in the UNC-CH cohort was slightly lower than that in the UPMC cohort (27% vs. 35%, respectively); this difference could have potentially influenced the histopathology of MBM (i.e., inflamed versus non-inflamed). However, systemic treatments following craniotomy may have more likely influenced disease-specific OS between the two cohorts. In the more contemporary UNC-CH cohort, 45% of patients received systemic therapies that were FDA-approved after 2011 in comparison to 11% of patients in the UPMC cohort. These effective immunotherapies and targeted therapies may have preferentially favored non-inflamed melanomas. However, exploratory analysis in the UNC-CH cohort suggests that patients with high density of TILs in MBM (H&E stain) who subsequently received immune checkpoint inhibitors had longer OS compared to all other patient subgroups.

Since our earlier report on gene expression profiling of same-patient, PrM, EcM, and MBM, we and others have developed robust immunogenetics methodologies to investigate and quantify the abundance of various immune cell subsets (27). We therefore felt that it was important to re-analyze these data, in particular from a patient cohort that had received little to no prior radiation (i.e., UPMC). Our analyses failed to identify any bone marrow-derived or resident glial populations with prognostic significance except for previously reported ones by us and others. However, our reanalysis of same-patient melanoma specimens that includes 26 EcM-MBM pairs showed that specific bone marrow-derived immune cell subsets, such as macrophages and γδT cells, may be uniquely abundant in MBM as opposed to EcM and PrM. In contrast with previous studies (31), we did not find differences in the gene expression scores of monocytic lineage cells, natural killer cells, and effector T cells in MBM compared with EcM. We do not attribute the differences between the two cohorts to the higher number of melanoma-infiltrated lymph nodes included in EcM compared to other cohorts, because these distinct immune cell subsets were not significantly different between EcM in lymph nodes versus EcM in non-lymph nodes. Furthermore, craniotomy specimens from the UPMC cohort had not previously received radiation, much like in previous studies (31, 37, 38). We confirmed that γδT cells are variably present in MBM. Our study is the first to report γδT cells’ presence in a solid metastatic tumor to the brain. γδT cells are unconventional T cells that can have diverse roles depending on the tumor microenvironment [reviewed in (39)]. We also found that not all MBM with TILs also had γδT cells and that the abundance of γδT cells was not prognostic. Nevertheless, the patient subgroup with present γδT cells in craniotomy specimens who subsequently received immune checkpoint inhibitors lived significantly longer compared to the patient subgroup who had absent γδT cells in craniotomy specimens and did not subsequently received immune checkpoint inhibitors. In summary, we identified that γδT cells are variably present in MBM. However, our results could not elaborate on the role —antitumor or protumor— of γδT cells in established MBM.

Only a handful of cancers have a high propensity to bleed if metastatic to the brain (10). Our study found that the resected brain lesion’s tumor size positively correlated with histopathologic evidence of hemorrhage. Although this is not a surprising finding, we also found that the degree of hemorrhage was inversely proportional to the density of mature vessels, namely endothelial cells (CD31+) surrounded by pericytes (αSMA+). This finding supports previous reports that blood vessels with deficient or absent pericyte coverage or mere abnormal association between pericytes, endothelial cells, and matrix are structurally deficient, leading to hemorrhage (40, 41). What may drive the formation of immature blood vessels that lack pericytes in MBM with high intratumoral hemorrhage is unclear. Of the two crucial angiogenic cytokines that we investigated in this study previously associated with sizeable abnormal blood vessels, only bFGF associated with hemorrhage. High bFGF expression by melanoma may suggest an overall aggressive potential for melanoma cells to form immature blood vessels (‘vasculogenic mimicry’) (42). In contrast, VEGF expression did not significantly associate with hemorrhage in MBM. The association between bFGF and intratumoral hemorrhage in MBM may also be clinically important given the availability of targeted therapies against the fibroblast growth factor receptors [reviewed in (43)].

Our analysis regarding the expression of angiogenic cytokines in the four more predominant compartments within MBM revealed that TILs and melanoma cells (and normal brain) express VEGF at comparable levels. Melanoma cells themselves can be a source of VEGF and bFGF, which may explain our earlier observation regarding the significant correlation between tumor volume from craniotomy and degree of peritumoral edema. These findings may have clinical implications (**Supplementary Figure 5**) (13). For example, VEGF’s similar expression by both TILs and melanoma cells may explain why single-agent PD1 inhibitors may increase peritumoral edema in untreated/active MBM (4, 37). The least frequent tumors that underwent craniotomy and had absent peritumoral edema had low/absent TILs. These tumors may express low VEGF levels, perhaps because there are no VEGF-producing TILs and because melanoma cells themselves may not express VEGF. In these cases, corticosteroids may not provide significant clinical benefit (but also not harm the host immune response either) since these tumors are not inflamed. On the other spectrum, the more abundant tumors with a high degree of vasogenic edema may express high VEGF levels predominantly from melanoma cells (and perhaps bFGF, among other angiogenic factors) because these tumors are infrequently inflamed. Corticosteroids would be extremely beneficial in these cases to treat peritumoral edema without a significant negative impact on host immune response. The most abundant tumors with present/low vasogenic edema were the ones more likely to be inflamed. For these tumors, the use of corticosteroids has more challenging tradeoffs balancing the need to reduce swelling with a higher likelihood to compromise the host immune response. For these tumors, steroid-sparing strategies that involve targeting VEGF, or even bFGF, may be more important to consider.

Our study has several limitations. The UNC-CH cohort is significantly smaller compared to the UPMC cohort, including the patient subset with high TILs and subsequent treatment with immune checkpoint inhibitors that trends to live the longest compared to all other groups. However, UNC-CH’s sample size is comparable to other previously reported craniotomy datasets (31, 37, 38). Furthermore, OS was the only clinical endpoint that was used in this study. Given that UPMC and UNC-CH cohorts demonstrate large differences in the type of systemic treatments that patients received following craniotomy, direct comparison between these two cohorts is challenging. In addition, interobserver variability may somewhat account for differences in the semiquantitation of histopathologic features, despite our efforts to rely on dichotomous variables that are more reliably reproducible. Finally, the tumor imaging (VEGF, bFGF, blood vessel type) and radiographic findings (peritumoral edema, tumor size) were only available for the UNC-CH cohort.

In summary, we have performed a comprehensive histopathologic and radiologic analysis of brain melanoma specimens that were collected from an independent patient cohort (UNC-CH). Patients with high TILs in craniotomy specimens who subsequently received immune checkpoint inhibitors trended to live the longest. Intratumoral hemorrhage, a significant clinical problem in MBM, is not merely associated with tumor volume but also with the density of immature blood vessels (CD31+αSMA-) and melanoma-derived angiogenic cytokines (e.g., bFGF) in MBM. The degree of peritumor edema, another significant clinical problem in MBM, is associated with the density of TILs. VEGF, the principle angiogenic cytokine that is associated with peritumoral, but not bFGF, can be expressed by TILs, in addition to melanoma cells themselves, suggesting that peritumoral edema is not driven by angiogenic cytokines solely produced by melanoma cells themselves but also by TILs, especially if it is low-grade. This finding implies that steroid-sparing treatments against the VEGF, but even against the bFGF pathway, may positively affect both host immune response and intratumoral hemorrhage. Specific immune cell subsets, such as γδT cells and macrophages may be more abundant in brain metastases than extracranial tumors.

## Data Availability Statement

The raw data supporting the conclusions of this article will be made available by the authors, without undue reservation.

## Ethics Statement

The studies involving human participants were reviewed and approved by Institutional Review Board, The University of North Carolina at Chapel Hill. Written informed consent for participation was not required for this study in accordance with the national legislation and the institutional requirements.

## Author Contributions

Conception or design of the work: DT, ME, MK, JK, BV, SM. Data collection: DT, SR, EB, AS, BM, FC, CL, G-SK, FA, DO, MK, NN-F, SM. Data analysis and interpretations: DT, ED, AS, ME, JK, BV, SM. Manuscript writing: DT, ED, AS, JK, BV, NN-F, SM. All authors contributed to the article and approved the submitted version.

## Funding

Supported by the University Cancer Research Fund and the National Cancer Institute Cancer Clinical Investigator Team Leadership Award (5P30CA016086-38, SJM).

## Conflict of Interest

The authors declare that the research was conducted in the absence of any commercial or financial relationships that could be construed as a potential conflict of interest.

## Abbreviations

Ang2, angiopoietin-2; bFGF, basic fibroblast growth factor; EcM, extracranial metastases; FFPE, formalin-fixed paraffin-embedded; GEE, Generalized estimating equations; HIF1α, hypoxia-inducible factor-1 alpha; H&E, hematoxylin and eosin; IF, immunofluorescence; IHC, immunohistochemistry; MBM, melanoma brain metastases; MM, metastatic melanoma; MRI, magnetic resonance imaging; OS, overall survival; PrM, primary melanoma; ROI, region of interest; TCR, T-cell receptor; TILs, tumor-infiltrating lymphocytes; UNC-CH, The University of North Carolina at Chapel Hill; UPMC, University of Pittsburgh Medical Center; VEGF-A, vascular endothelial growth factor-A; α-SMA, alpha-smooth muscle actin.
