# Supplementary material for: Brain Tumor Microenvironment and Angiogenesis in Melanoma Brain Metastases
Source: Front Oncol. 2021 Jan 21;10:604213. doi: 10.3389/fonc.2020.604213 (PMC7860978; doi:10.3389/fonc.2020.604213)
Supplement: Supplementary file 1 [file DataSheet_1.docx]

**Supplementary Figure 1. Stepwise approach to develop gene signatures for human neurons, oligodendrocytes, astrocytes, and microglia.**

**Supplementary Figure 2. Prognostic significance of various gene expression signatures in melanoma brain metastases.** Signatures are grouped according to immune cell subtypes, cell processes and resident brain cells. Abbreviations: Th, T helper; Tcm, central memory T cells; Tem, effector memory T cells; TFH, follicular helper T cells; Tgd, gamma delta T cells; Treg, regulatory T cells; LCK, lymphocyte-specific protein tyrosine kinase; DC, dendritic cells; aDC, activated DCs; iDC, immature DCs; pDC, plasmacytoid DCs; EMT, epithelial-to-mesenchymal transition.
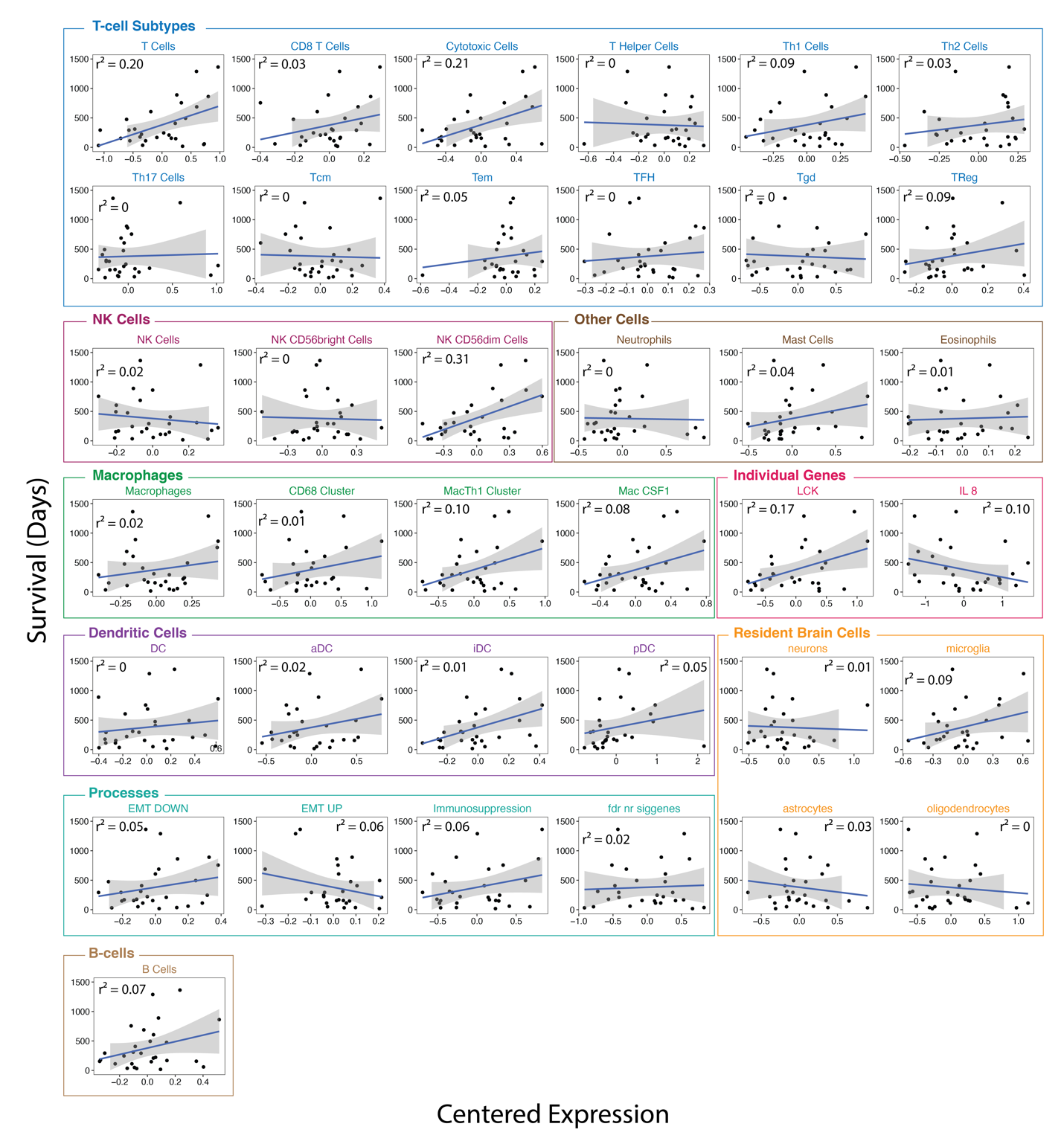


**Supplementary Figure 3. Forest plots showing gene signatures with prognostic significance from the UPMC craniotomy cohort (N=29).** Survival analysis was conducted using a univariate Cox proportional hazards regression model. Each gene signature was used as a covariate in the model, and survival was defined as days from craniotomy until death from metastatic melanoma.

**
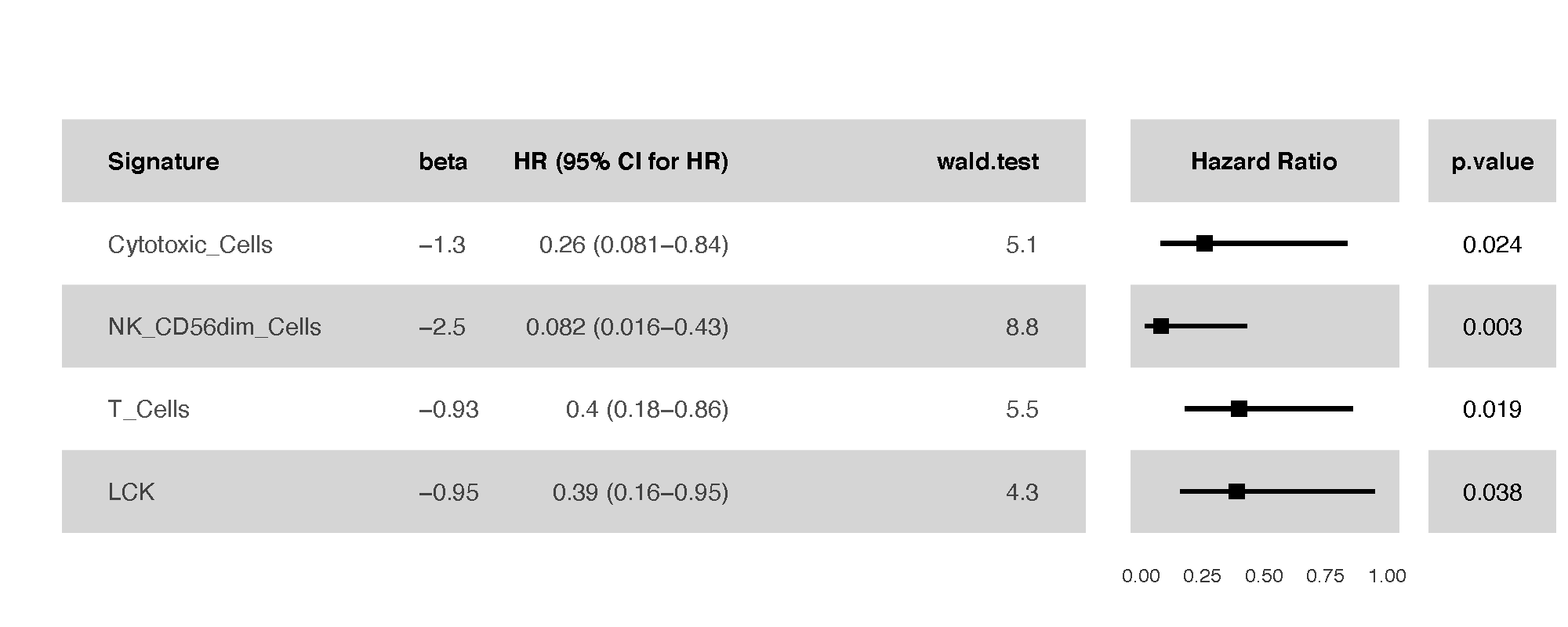
**

**Supplementary Figure 4. Abundance of various immune cell subsets in extracranial metastases located in lymph nodes versus non-lymph nodes in metastatic melanoma (UPMC cohort).** Signatures are grouped according to immune cell subtypes. * adjusted p-value <0.05. Abbreviations: Th, T helper; Tcm, central memory T cells; Tem, effector memory T cells; TFH, follicular helper T cells; Tgd, gamma delta T cells; Treg, regulatory T cells; LCK, lymphocyte-specific protein tyrosine kinase; DC, dendritic cells; aDC, activated DCs; iDC, immature DCs; pDC, plasmacytoid DCs;

**Supplementary Figure 5. Proposed model that summarizes association between peritumoral edema, intratumoral hemorrhage, tumor-infiltrating lymphocytes, blood vessel density and type, and the two principle angiogenic factors in patients with melanoma brain metastases.**


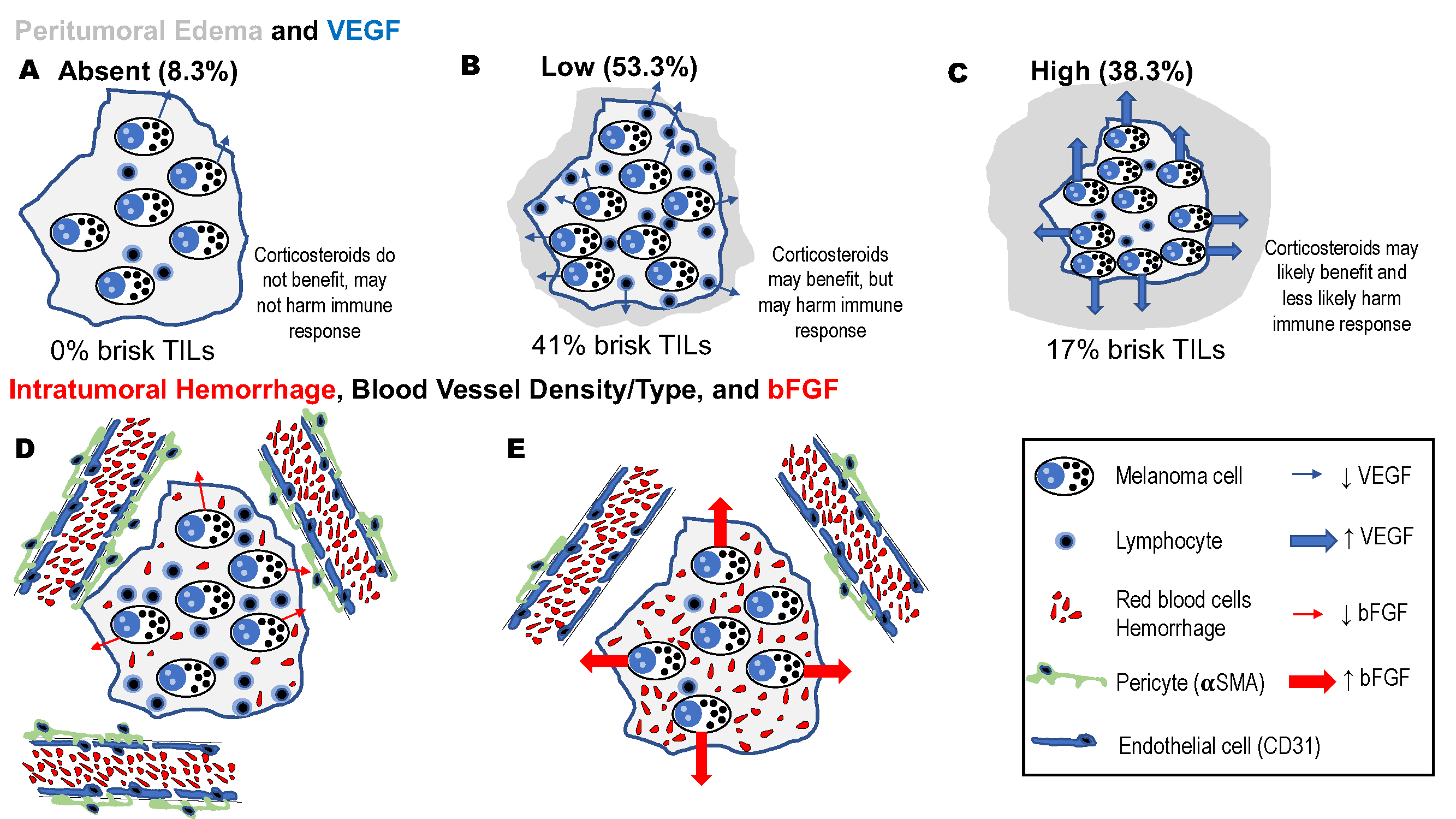


**Supplementary Table 1.** List of genes that comprise the macrophage gene signature.

**ENTREZ ID. SYMBOL**

1      8685   *MARCO*

2      6374   *CXCL5*

3      6447    *SCG5*

4      6819 *SULT1C2*

5      1513    *CTSK*

6     81035 *COLEC12*

7     26577 *PCOLCE2*

8      1118   *CHIT1*

9      5730   *PTGDS*

10     3730   *ANOS1*

11    23601  *CLEC5A*

12     2239    *GPC4*

13     4199     *ME1*

14    58511 *DNASE2B*

15     6354    *CCL7*

16     2335     *FN1*

17     2760    *GM2A*

18      950  *SCARB2*

19      586   *BCAT1*

20    26064   *RAI14*

21     4481    *MSR1*

22     1296  *COL8A2*

23     9332   *CD163*

24      348    *APOE*

25     1116  *CHI3L1*

26    10533    *ATG7*

27     8832    *CD84*

28     2230    *FDX1*

29    51338  *MS4A4A*

30   259230   *SGMS1*

31     2012    *EMP1*

32     1536    *CYBB*

33      968    *CD68*
